# Hesitant and anti-vaccination groups: A qualitative study on their perceptions and attitudes regarding vaccinations and their reluctance to participate in academic research- an example during a measles outbreak among a group of Jewish parents in Israel

**DOI:** 10.3389/fpubh.2022.1012822

**Published:** 2022-11-09

**Authors:** Rana Hijazi, Anat Gesser-Edelsburg, Paula Feder-Bubis, Gustavo S. Mesch

**Affiliations:** ^1^School of Public Health, University of Haifa, Haifa, Israel; ^2^Head of the Health Promotion Program and Head of the Health and Risk Communication Lab, School of Public Health, University of Haifa, Haifa, Israel; ^3^Department of Health Policy and Management, Faculty of Health Sciences and Guilford Glazer Faculty of Business and Management, Ben-Gurion University of the Negev, Beersheba, Israel; ^4^Department of Sociology, University of Haifa, Haifa, Israel

**Keywords:** vaccine hesitancy, health communication, vaccines, Israel, qualitative study

## Abstract

**Background:**

Vaccination is widespread in Western countries and, overall, there is a high vaccination rate. However, immunization is still an enduring challenge. In recent years, the number of parents who choose to delay or refuse vaccines has risen.

**Objectives:**

(1) to identify the perceptions and attitudes of hesitant and anti-vaccination parents regarding vaccination in general, and vaccinating their children in particular and; (2) to describe the responses of potential participants to the request to participate in academic research regarding their perceptions and attitudes on the subject of vaccines.

**Methods:**

The research employs the qualitative hermeneutic phenomenological method using two research tools: (1) in-depth interviews with 7 hesitant and 11 anti-vaccination Jewish parents in Israel; and (2) the researchers' field notes from this study process, which describe the responses of 32 potential participants to the request to participate in this academic research.

**Results:**

The main findings indicate that while most of the interviewees admit to the efficacy of vaccines in preventing diseases, they oppose the way in which vaccines are promoted—based on providing partial information and disregarding parents' concerns and questions. Therefore, they demand transparency about the efficacy and safety of vaccines. The findings also point to a paradoxical finding. On the one hand, these groups claim that health organizations do not understand their position, referring to them as “science-deniers”, even though they are not. On the other hand, these parents choose to refrain from participating in scientific studies and voicing their opinions, thereby perpetuating the situation of being misunderstood.

**Conclusion:**

Hesitant and anti-vaccination groups express mistrust in academic institutions and health organizations. Therefore, an effective dialogue that would include hesitant and anti-vaccination groups, the academy, and health organizations may contribute to a better understanding of the barriers that prevent these groups from getting vaccinated or vaccinating their children and promote public health.

## Introduction

Vaccination has been one of the most effective medical interventions to reduce death and morbidity caused by infectious diseases (1–3). While access to immunization is an enduring challenge, acceptance of and agreement to be vaccinated also remains an issue of great importance, and is affected by the individual's feelings, attitudes, and beliefs about vaccination (4). In recent years, the number of parents and individuals choosing to delay or refuse vaccines has risen (2, 5–7). Although there is generally a high rate of vaccination coverage in most developed Western countries, there is a growing number of individuals who express doubts and concerns about vaccination, also among parents who choose to vaccinate their children (8). Hesitant individuals who have questions and concerns have been shown to have lower levels of vaccination uptake (9), which may substantially impact vaccination coverage and increase the risk of outbreaks (10). By being vaccinated individuals are getting protected from being infected themselves and they also cannot pass this infection on to other people. However, to achieve herd immunity, a large proportion of the population needs to be vaccinated. This proportion varies depending on the germ and how contagious it is. Suboptimal vaccination coverage prevents herd immunity from being reached and extends susceptibility to vaccine-preventable diseases (11). Therefore, the World Health Organization (WHO) recognizes vaccine hesitancy as one of the 10 threats to global health (12), and recommends scaling up advocacy efforts to improve understanding of the value of vaccines and to allay fears leading to vaccine hesitancy (13). There is a wide spectrum of attitudes toward vaccination, including those who are pro-vaccination and accept all vaccines; those who are hesitant and have many concerns, but may entirely or partially vaccinate; and those who refuse all vaccines (6).

Several explanatory models were developed to elucidate the determinants of vaccine hesitancy. For example, The World Health Organization's (WHO) Strategic Advisory Group of Experts (SAGE) developed the “3Cs” model, which is based on three primary components: confidence in vaccines, complacency (low perceived usefulness of vaccination), and convenience (perceived constraints to accessing health or vaccination services) (14, 15). Another model developed by SAGE is the “Vaccine Hesitancy Determinants Matrix”, which categorizes the reasons for hesitancy into three categories: contextual, individual and group, and vaccine/vaccination-specific influences (1). In addition, a conceptual model, adapted from a schema summary of discussions held during a workshop on the cultural and religious roots of vaccine hesitancy in Canada, illustrates that vaccine hesitancy at an individual level is influenced by a range of factors such as knowledge or experience; and historical, political, and socio-cultural influences (16).

All the models assume that vaccine hesitancy is affected by trust in health care professionals, the health care system, science, and socio-political context. Trust can be defined as “a relationship that exists between individuals, as well as between individuals and a system, in which one part accepts a vulnerable position, assuming the competence of the other, in exchange for a reduction in decision complexity” (17), such as providing information on the advantages and disadvantages of decisions. Trust is not uniform across all vaccines and may vary for different vaccine components (e.g., multiple antigens, adjuvants), for new vs. “old” vaccines, and according to past or present vaccine-associated controversies. Trust also depends strongly on patients' relationship with the healthcare professionals involved in vaccination: patients often seek a professional compatible with their values and beliefs; their trust is a precondition for the delegation of immunization and other health care decisions (16, 18, 19). In addition, a consensus between governments, their scientific advisors, and the public is crucial to sustaining effective and consistent communication. This consensus is built on mutual trust between scientists and policymakers, and between governments and their citizens (20). Studies have shown that clear and constant communication by public health officials and government spokespersons affects public trust and increases the likelihood of people complying with recommendations and protective behaviors like vaccination (21, 22). Furthermore, inconsistent communication may decrease the public's trust in the government's ability to manage a crisis effectively, and increase skepticism about public health recommendations (23, 24).

Most of the scientific literature has focused on vaccine hesitancy rather than anti-vaccination. In the context of hesitancy, most studies have developed campaigns that can predict and measure hesitancy or persuade hesitant groups. For the authorities, understanding the attitudes that underline vaccination refusal and hesitancy is essential for predicting vaccination behavior and developing vaccination-promoting campaigns. Some studies focus on measuring vaccine hesitancy or anti-vaccination (25). Others consider hesitant and anti-vaccination groups a threat to people's health and herd immunity, and recommend developing strategies for debunking the myths peddled by the anti-vaccination movement and even introducing legislation that promotes or mandates vaccination (26). Recent systematic reviews focus on understanding the psychological factors that motivate people to reject the science in vaccinations (27, 28), analyzing anti-vaccine messages in social media (29), and identifying existing narrative interventions aimed at countering anti-vaccination conspiracy theories (28). In addition, a recent systematic review indicates a disconnect between the current vaccine hesitancy and the broader health-related trust literature (17).

However, few studies have focused on the anti-vaccination group and have mainly tried to understand and explore the history of the development of the anti-vaccination movements. The history of anti-vaccination, identified by medical anthropologist Anna Kata, includes questioning the safety and efficacy of vaccines, promoting alternative cures, claiming that vaccination infringes on individuals' civil liberties and asserting that vaccines are immoral (30). Anti-vaccination advocates typically represent well-organized entities with explicit agendas, ranging from financial interests (selling alternative cures) to ideological or political commitments (opposing specific legislation). Larson and colleagues suggest that organizations also frequently shift their goalposts, claiming that vaccines cause any number of maladies while supporting opposing political platforms, and that these themes are widespread on social media (31).

According to the above, most literature to date has referred to the hesitant group in order to characterize them and examine effective strategies to promote vaccination. Still, at the same time, it appears that it has failed to understand them and engage in dialogue with them. Therefore, this study aims to expose not the point of view of organizations toward the hesitant and anti-vaccination groups, as most literature discusses, but to focus on hesitant and anti-vaccination parents and how they perceive the health system and its attempt to communicate with them. This is a pioneering study as, to the best of our knowledge, almost no research to date has been conducted from the point of view of the opposing and hesitant parents.

This study seeks to provide a deeper understanding of the attitudes and perceptions of hesitant and anti-vaccination parents from their point of view, and aims to (1) identify the perceptions and attitudes of hesitant and anti-vaccination parents regarding vaccination in general, and vaccinating their children in particular and; (2) describe the parents' responses to the request to participate in academic research about their perceptions and attitudes regarding vaccination.

## Methods

### Research design and procedure

The research was conducted in Israel during a measles outbreak. The overall vaccination coverage rate in Israel is high (32, 33). However, parental hesitancy regarding recommended childhood vaccines has become more prevalent in Israel as in other countries in recent years (32–35). A recent assessment shows that 7.5%-9% of parents do not adhere to the routine vaccination schedule (36, 37). Vaccination compliance in Israel was found to rely on personal risk-benefit perceptions (38). Vaccine hesitancy in Israel is associated with higher education, indicating that hesitancy is a result of informed decision-making, rather than ignorance (37, 39). Elran and colleagues found that parents' decision to vaccinate their children is most influenced by their degree of trust in information sources about vaccination, particularly the nurses at the Mother and Child Health Clinics (32).

This research is based on a qualitative, hermeneutic phenomenological methodology as developed by Martin Heidegger (40). Heidegger proposed that humans are actors in the world. He focuses on the relationship between an individual and his/her lifeworld (41). Therefore, to investigate any individual or group in an in-depth manner, the researcher must first gain access to their lifeworld. This enables the researcher to share this world with others and to further explore what it means—socially, politically, and historically—to be a part of this lifeworld (41–43). Hermeneutic phenomenology seeks “to understand the deeper layers of human experience that lay obscured beneath surface awareness and how the individual's lifeworld, or the world as he or she pre-reflectively experiences it, influences this experience” (42). Heidegger's approach was used in this study to reveal new insights into hesitant and anti-vaccination participants' experiences of the vaccination process in Israel, including social and political aspects, and particularly regarding their experience of how health organizations and the community perceive them.

We conducted in-depth interviews with 18 hesitant and anti-vaccination parents in Israel during a measles outbreak. The qualitative approach enables the researcher to examine the perceptions and attitudes of the research populations from the perspective of the individuals themselves. In addition, the use of qualitative, in-depth interviews stems from the difficulty of reaching the target audience of hesitant and anti-vaccination groups, and the subsequent difficulty of understanding these groups' attitudes and perceptions (44).

The study was approved by the the Faculty of Social Welfare and Health Sciences Ethics Committee for research with human subjects at the University of Haifa (Approval no. 421/17).

### Sampling and data collection

The interviews were conducted by phone during a measles outbreak in Israel from April 2019 until August 2020. Although the study was conducted partly during the COVID-19 pandemic, most of the interviews were conducted before the COVID-19 outbreak in Israel, and only two interviews were conducted during the COVID-19 outbreak in Israel. Therefore, the interviewees were not asked about the COVID-19 virus and its vaccine. However, the interview guide focused on routine vaccination in Israel in general and the participants' experiences of the vaccination process in Israel. The duration of each interview was approximately half an hour.

In the first stage, the researchers performed a purposeful criterion intensive sampling of hesitant and anti-vaccination Jewish parents of children under 12 years of age, who agreed to participate in the study. We chose parents with children under 12 years of age because the recommended routine vaccination schedule in Israel is intended for children up to 12 years of age. Only parents who met the inclusion criteria were included in the study. According to Patton, purposeful sampling is “a technique widely used in qualitative research for the identification and selection of information-rich cases for the most effective use of limited resources” (45–47). Relevant participants were selected to supply as much knowledge as possible. In addition, the sample aimed to provide the maximum variation in order to reflect a wide variety of hesitant and anti-vaccination parents with regard to the parents' place of residence in Israel, education, and the children's age (47).

We approached potential participants through parents' groups on WhatsApp and Facebook. The groups were classified by the researchers as anti-vaccination and hesitant groups due to their self-identification and content. The researchers posted a post about the research and its goals on these groups, and invited parents to participate. Parents who agreed to participate, after receiving an explanation about the study and its objectives, were asked to contact the researchers and schedule a telephone interview.

To enlarge our sample, we proceeded to perform snowball sampling. Parents who had already participated in the study were asked to assist in identifying other potential interviewees. The researchers approached 10 potential interviewees, who agreed to participate in the study.

The researchers initially approached a total of 32 parents to participate in the study in the two sampling stages. A total of 18 out of the original 32 agreed to participate in the entire interview and completed the whole study process (see Table 1). The other 14 parents refused to be interviewed, but agreed to explain why they did not want to participate in the study. The Results section presents the interview findings, and details the reasons for parents' general reluctance and, finally, refusal to complete the full interview.

**Table 1:** Sociodemographic characteristics of the interviewees (*N* = 18).

		**Hesitant (*****N*** = **7)**		**Anti-vaccination (*****N*** = **11)**		**Total (*****N*** = **18)**	
**Variables**		**Frequency**	**Percentage (%)**	**Frequency**	**Percentage (%)**	**Frequency**	**Percentage (%)**
Gender	Male	1	14.3	3	27.3	4	22.2
	Female	6	85.7	8	72.7	14	77.8
Age (years)	30–35	4	57.1	3	27.3	7	38.8
	36–40	2	28.6	3	27.3	5	27.8
	41–45	1	14.3	2	18.1	3	16.7
	46–50	-	-	3	27.3	3	16.7
Ethnicity	Jewish	7	100.0	11	100.0	18	100.0
Marital status	Single parent	-	-	1	9.1	1	5.5
	Married	6	85.7	8	72.7	14	77.8
	Widow	-	-	1	9,1	1	5.5
	Parent in a relationship	1	14.3	1	9.1	1	5.5
Education	Primary school	-	-	1	9.1	1	5.5
	Secondary	-	-	1	9.1	1	13.1
	BA	4	57.1	5	45.4	9	50.0
	MA	3	42.9	3	27.3	6	33.3
	PhD	-	-	1	9.1	1	5.5
Occupation/ profession	Self-employed	-	-	2	18.2	2	11.1
	Health worker	3	42.9	2	18.2	5	27.8
	Education	1	14.2	1	9.1	2	11.1
	Engineering	-	-	2	18.2	2	11.1
	Unemployed	-	-	1	9.1	1	5.6
	Other	3	42.9	3	27.2	6	33.3
Total		7	38.9	11	61.1	18	100.0

The interviews were conducted in Hebrew (the participants' first language) and audio-recorded. Then, they were transcribed verbatim and analyzed. Moreover, the recruitment process for each of the 32 potential participants we approached was recorded in the field notes. This includes their responses to the request to participate in an academic study, why they refused to participate, and how many times the researchers contacted them. Potential participants who refused to participate in the study were asked about the reasons behind their reluctance to participate in an academic study. Only reluctant participants who gave their consent to use their answers were included in this study.

### Research tools

This study is based on two research tools. In-depth interviews were conducted based on the following interview guide (see Table 2). The interview guide questions were developed based on the scientific literature about vaccine hesitancy and anti-vaccination and the researchers' previous studies on vaccine hesitancy (48, 49).

**Table 2:** Interview guide questions.

**Question topics**	**Questions**
**Warm-up questions**	• Can you please tell me about your lifestyle, with a focus on health-related issues?. • How do you keep your children healthy?.
**Emotions toward vaccines**	• What comes to mind when you hear the word “vaccination" or “vaccines”?. • How do you feel when you hear the word “vaccine” (e.g., safe, concerned, etc.,)?.
**Attitude toward vaccination**	• What is your general attitude toward vaccinating your children (Do/Have you vaccinate/d your children with all the routine vaccines, hesitate/d to vaccinate regarding a specific vaccine, or do you not vaccinate at all)?. • Has your attitude toward vaccines changed over the years?. • Did your attitude change from the first child to the second?. • Follow-up question: What are the reasons for this?.
**Knowledge about vaccines, measles and MMR or MMRV vaccine against measles**	• What did you know about vaccines during the decision-making process?. • What was most important for you to know about vaccines?. • In your opinion, what is the aim of vaccines?. • How do you think vaccines work inside the body?. • How do vaccines prevent disease?. • What are the advantages or benefits of vaccines?. • What are the risks of giving vaccines in general, or certain vaccines in particular?. • What do you know about measles?. • Have you vaccinated your children with the MMR vaccine?. • What do you know about the vaccine against measles?.
**Risk perception and self-efficacy regarding their children contracting the disease**	• Do you feel that your children are at risk of contracting measles and, if so, how do you protect your children from contracting measles?. • Do you feel that you have the necessary tools needed to protect your children from becoming infected with measles?.
**Information sources**	• Do you spend a lot of time searching for information on health issues? On what subjects?. • Do you spend a lot of time looking for information on vaccines?. • Do you spend a lot of time looking for information on measles and the vaccine against it?. • What information would you like to know about vaccines?. • What information would you like to know about measles and the vaccine against it?. • Where do you usually search for information?. • Do you feel you have the necessary tools needed to help you locate the information you are looking for?.
**Perceived trust in the Ministry of Health, other health system organizations, and public health officials**	• What is your level of trust in the Israeli health system (medical institutions and health workers)? Please elaborate and explain why. • Do you regard the Ministry of Health as a reliable source of information?. • Has the Ministry of Health ever provided information that answers your questions or concerns on a particular subject? Please elaborate. • Do you think you receive reliable and comprehensive information from the Ministry of Health?. • If not, how would you suggest the Ministry of Health improve the quality of the information provided?. • What factors affect the public's trust in the health care system in Israel (medical institutions and health workers)?. • What was your impression of the Ministry of Health's response during the measles crisis?. • What would you have advised the Ministry of Health to do during the measles outbreak in Israel?. • What information would you have advised the Ministry of Health to share with the public?. • Have you been exposed to information about the measles outbreak in Israel originating from the Ministry of Health?. • What information did you receive from the Ministry of Health?. • Do you feel that the Ministry of Health gave you reliable information about the measles outbreak in Israel?.
**Misinformation and uncertainty in the social media**	• Are you active on social networks? On which platforms and groups?. • Are you exposed to discourse on health issues? In what subjects?. • Have you been exposed to a debate about vaccines or measles on social media?. • Were you exposed to misinformation or uncertainty regarding health issues in social media and how has this information affected your attitudes and perceptions?.

The second research tool was the researchers' field notes (50, 51). In view of the study's second objective, the field notes describe the recruitment process in detail, including the difficulties of reaching the study population, the responses of the potential interviewees for the request to participate in the study, how many times the researchers contacted the potential interviewees, how much time passed until the interview was conducted, and the reasons why reluctant interviewees refused to participate in the study.

### Credibility and validity

The information was accessible to the researchers, and all steps in the research process were transparent, as were the analytic methods and the interpretations of the findings (52).

Since the interviews were conducted, and the data analyzed by a single researcher, the data results and interpretations were discussed between the two main researchers during peer debriefing sessions (53). When there was disagreement about results interpretation and coding, this was discussed until a consensus was reached. Elaborated and detailed transcripts and written field notes increased study *dependability* (44, 54).

### Analysis

Data were analyzed using thematic analysis (55). The transcripts were read and re-read, noting down initial ideas separately by two of the researchers. Then, initial codes were generated systematically across the entire data. The next step was extracting potential themes by looking over the codes we have created and gathering all data relevant to each potential theme. Specifically, we focused on the participants' attitudes toward vaccination, dilemmas in vaccination decision-making, concerns regarding vaccination, and the linkage between intention and behavior. Lastly, sub-themes were integrated into primary and secondary themes. Explanations and inferences are presented in the Results section, accompanied by quotes from the interviewees' statements.

Moreover, the field notes were analyzed by the researchers using conventional content analysis (56). We focused on refusing to participate in an academic study, identifying their reasons for not participating, and their trust in academic research. The researchers read all the data several times while focusing on the data about reluctant participants. Then, an initial analysis was conducted to derive recurring codes that identify the reasons for refusing to participate in an academic study. Subsequently, the researchers approached the text by making notes of their initial analysis, followed by labels for codes. Codes were then organized into two main categories and presented in the Results section.

## Results

The results are divided into two parts following the study's objectives: (1) According to the first study objective, seven themes arose from the analysis of the interviews regarding hesitant and anti-vaccination parents' perceptions and attitudes and how the health authorities and the community perceive them. (2) According to the study's second objective, two themes arose from the analysis of the responses of hesitant and anti-vaccination parents to participate in academic study requests.

### The first study objective: Hesitant and anti-vaccination parents' attitudes regarding vaccination in general, and vaccinating their children in particular from their perspective

This part of the results consists of seven themes and focuses on hesitant and anti-vaccination parents' perspectives of how they perceive vaccines, what they think about the vaccination process, and the way the health authorities perceive them (see Table 3).

**Table 3:** Themes and sub-themes.

**Themes**	**Sub-themes**
1. Not “against” vaccines and vaccination, per se	
2. How health organizations communicate vaccine information to the public	2.1. Lack of transparency and ineffective communication between health organizations and the public 2.2. The strategic use of fear appeals
3. Vaccination process management by health organizations	3.1. Vaccination coercion vs. autonomy 3.2. Authorities' health decisions are motivated by conflicts of interest
4. Generic vaccination process vs. personalized vaccination	4.1. A tailored vaccination schedule 4.2. Splitting up vaccines
5. Persecution of anti-vaccination parents and dividing the community	
6. Vaccines' effectiveness and safety	6.1. Carrying out studies to test the effectiveness and safety of vaccines 6.2. Ignoring vaccine-related damages and reports on adverse effects
7. Correcting misinformation and communicating uncertainty on social media	

#### Not “against” vaccines and vaccination, per se

Contrary to the popular belief that anti-vaccination individuals deny the effectiveness of vaccines, this study shows the opposite. Many of the interviewees (5 hesitant and 5 anti-vaccination) believe in the efficacy of vaccines in preventing diseases and their vital historical role in eradicating diseases.

“The benefits of vaccines historically in eradicating diseases is clear to me.” (Interviewee 5).

However, they oppose the way in which vaccination is promoted because they don't believe in the necessity of all the given vaccines recommended by the Ministry of Health:

“The primary goal was to eradicate serious diseases that existed in the world, for which there was no treatment or not enough knowledge about their treatment. I think the primary goal of vaccines is good...When people started developing vaccines, they had a specific purpose in mind – to help the public.” (Interviewee 15).

Instead, participants noted that vaccines should be given on an “as needed” basis (for example: at the time and place of a disease outbreak, when there is a real and present risk of infection, etc.,).

“During an epidemic outbreak, it is advisable to vaccinate only those populations that are at risk. For example, during a measles outbreak, only the population living in an area where there is an active outbreak should be vaccinated.” (Interviewee 17).

#### How health organizations communicate vaccine information to the public

The ways in which health organizations communicate vaccine information to the public emerged as one of the main themes in this study. The interviewees described a lack of transparent communication by the Israeli Ministry of Health to the public, and emphasized the use of fear appeals to motivate the public to get vaccinated. Therefore, this main theme consists of two sub-themes: (1) a lack of transparency and ineffective communication between health organizations and the public; and (2) the strategic use of fear appeals.

##### Lack of transparency and ineffective communication between health organizations and the public

Thirteen out of 18 parents (5 hesitant and 8 anti-vaccination parents) mentioned that the Israeli Ministry of Health does not provide complete and accurate information in general, and regarding vaccines, specifically. Therefore, they demand transparency and providing the public with complete and accessible information.

“Explain the instructions [regarding vaccination] precisely and the rationale behind these instructions, instead of just giving instructions… explain why these instructions are given, so that people can understand, and provide reliable information sources, so that whoever wants to know where to find the information and understand the rationale behind it, can do so…The Israeli Ministry of Health needs to be as transparent as possible.” (Interviewee 7).

According to these participants, the Israeli Ministry of Health provides partial information in an attempt to influence the public's decision-making process and motivate them to get vaccinated and vaccinate their children. Moreover, the interviewees mentioned that the information provided by the Israeli Ministry of Health does not address their concerns or answer their questions, but instead ignores public inquiries and concerns.

“The parents are given minimal information, unless they ask or investigate, as if to say: “You don't need to know; don't be confused by the facts, you're just parents.” Thus, the level of information varies greatly from nurse to nurse at publicly funded Family Care Centers.” (Interviewee 1).

The interviewees also expressed difficulty finding the information they were looking for concerning health issues and vaccination, describing it as inaccessible and unavailable. Only interviewees with a high level of education expressed high self-efficacy in searching for information and finding answers to their questions.

“I have degrees; I know how to look for information. I am very good at searching for information. I have patience. I know that my English is at the mother tongue level. I know how to read scientific studies and glean insights. But not everyone is like that, so transparency should be much higher. And accessibility to the information should be much higher.” (Interviewee 15).

Consequently, they demand complete transparency to all the information when it comes to the process of decision-making regarding their health.

“… There's no need to hide anything. Even if an epidemic, disease, or problem breaks out, the public should be informed about everything …Everything should be shared with the public.” (Interviewee 17).

##### The strategic use of fear appeals

Many of the interviewed parents (2 hesitant and 8 anti-vaccination) said that the primary strategy used by health authorities to promote vaccination is fear appeals. Participants stated that the purpose of this strategy is to generate “hysteria” among the public regarding the severity of the disease and the consequences of non-vaccination. They also described this strategy as ineffective.

“The Ministry of Health's strategy of intimidation doesn't work. Even those who study marketing know that intimidation only works to a certain extent.” (Interviewee 3).

“I didn't like all the hysteria. I think the goal was probably to motivate more people to get the vaccine, and I think it worked. So, maybe according to the Ministry of Health, they succeeded.” (Interviewee 18).

#### Vaccination process management by health organizations

Vaccination process management by health organizations emerged as a main theme in this study. This theme consists of two sub-themes: (1) vaccination coercion vs. autonomy; and (2) authorities' health decisions are motivated by conflicts of interest.

The interviewees described how the Israeli Ministry of Health manages the vaccination process, including the motives and interests behind the decision-making process regarding vaccination, and how the autonomy principle is violated by presenting vaccination as obligatory although it is actually voluntary.

##### Vaccination coercion vs. autonomy

Five parents (2 hesitant and 3 anti-vaccination) expressed concern with what they perceived as vaccination coercion. They claimed that even though vaccination in Israel is voluntary, it is presented as being mandatory. Consequently, vaccination obligation is slowly being reintroduced.

“The growing concern is the issue of forcing people to vaccinate, and lobbyists who seek to make vaccination mandatory, which is a violation of the individual's rights and freedom. This is the real struggle.” (Interviewee 8).

The interviewees also believe that the principle of autonomy should not be violated. In addition, they feel that the individual's decision regarding vaccination should be entirely their own; instead, the health authorities make the decision for everyone. Therefore, the interviewees suggest that the parents should make an informed decision regarding vaccination, based on complete and transparent information from the health authorities.

“If someone gets vaccinated, I don't say anything…everyone makes the best and right choices for their children. Everyone has their own considerations. And if you do choose to vaccinate your children, then that's the best thing for your children.” (Interviewee 11).

##### Authorities' health decisions are motivated by conflicts of interest

Most of the interviewees (5 hesitant and 10 anti-vaccination) believe that the authorities' decision-making process is motivated by a conflict of interests and not only public health interests.

“In general, I don't think the Ministry of Health wants to kill us. But I do think that sometimes there are other interests that aren't in the public's best interest.” (Interviewee 10).

For example, they believe that the authorities' decision-making process regarding vaccination is motivated by political and economic interests. These interests represent the shared interests of pharmaceutical companies and governments.

“I believe the power of lobbyists and pharmaceutical companies is too great, and very suspicious…Doctors can't just come out and say things against vaccines… I watched YouTube videos about doctors from the United States, who talked about how their lives had been threatened because of the studies they'd published.” (Interviewee 13).

#### Generic vaccination process vs. personalized vaccination

Generic vaccination vs. personalized vaccination consists of two sub-themes: (1) a tailored vaccination schedule; and (2) splitting up vaccines. The interviewees suggested that the routine vaccination schedule should be modified according to the individual's needs, or the population's needs. They also suggested a personalized vaccination schedule, which is tailored according to the individual's health status and needs. In addition, some interviewees stated that they would agree to give their children certain vaccines if they were not given together with other vaccines.

##### A tailored vaccination schedule

Seven out of 18 parents (5 hesitant and 2 anti-vaccination) suggested that the vaccination schedule should be changed and adapted according to two levels of needs: individual needs and population needs. At the individual needs level, they stated that a vaccination schedule should be determined according to the child's needs and health status. Not all children need to receive all the vaccines. Some also recommended consulting an expert about which vaccines should be received and when to vaccinate.

“In a conversation with a representative from the Ministry of Health, she correctly said that this was a “recommendation”, and her aim was to achieve the highest average in Israel. Vaccines are not necessarily suitable for every child, just as any treatment is not suitable for every person.” (Interviewee 1).

At the population level, they asked questions regarding the legitimacy and purpose of mass vaccination. Some suggested that the routine vaccination schedule should be updated and changed because it is not adapted to our current daily lives. They explained that some vaccines should only be used in developing countries or countries that still experience outbreaks of these diseases.

“…Polio, for example. I think that only populations in third-world countries who are less hygienic, less healthy, and at a higher risk of becoming infected should be vaccinated.” (Interviewee 2).

##### Splitting up vaccines

Giving combination vaccines arose as a concern and a reason that prevents parents from vaccinating their children. Five out of 18 parents (2 hesitant and 3 anti-vaccination parents) claimed that they would agree to inoculate their children with certain vaccines, but not if they were part of combination vaccines.

“There are vaccines, for example, that are only given as combination vaccines. For example, I have no problem with the Tetanus vaccine. But this vaccine is given together with a Diphtheria vaccine and a Pertussis vaccine.” (Interviewee 1).

#### Persecution of anti-vaccination parents and dividing the community

Persecution of anti-vaccination parents is a theme that was mentioned by 6 parents (3 hesitant and 3 anti-vaccination). They described the discourse around vaccination on social media platforms as violent and aggressive. They also claimed that some health organizations, experts, and pro-vaccination individuals incite intolerance against anti-vaccination and hesitant groups.

“The discourse between pro-vaccination and anti-vaccination individuals on social media is very violent. I felt like if I told someone on the street that I don't vaccinate my child, they might just kill me. That's what the media and social networks have led to. The hand is very light on the keyboard. Many doctors on the net incite against anti-vaccination groups, mainly in this matter of dealing with diseases. They say there is no such thing as vaccine-related side effects, that everything has been studied, and you have to trust the authorities.“ (Interviewee 10).

In addition, the interviewees described the consequences of the violent discourse around vaccination as causing religious and social intolerance to vulnerable populations, such as ultra-Orthodox populations or low socio-economic populations.

“They divided the community by inciting religious and social intolerance; for example, by starting up with the ultra-Orthodox population because they do not vaccinate. They also incite against low socioeconomic status groups, although anti-vaccination parents do not belong to this population. This incitement is just terrible.” (Interviewee 1).

As a result of expressing an anti-vaccination attitude or questioning a vaccine's efficacy and safety, some of the interviewees have been personally attacked, harassed, or intimidated online.

“No parent would put themselves under attack and harassment. I received comments like: “Do not breed,” “Let the Welfare Services take your children…” (Interviewee 8).

#### Vaccines' effectiveness and safety

The interviewees focused on two sub-themes: (1) questioning the effectiveness and the safety of vaccines; and (2) ignoring vaccines' related injuries and reports of adverse effects.

##### Carrying out studies to test the effectiveness and safety of vaccines

Seven interviewees (1 hesitant and 6 anti-vaccination) claimed that there is a lack of studies proving and ensuring the efficacy and safety of vaccines. Therefore, they want more studies to be carried out in the future, such as prospective, long-term studies that compare vaccinated children and unvaccinated children.

“There is an utter lack of studies comparing vaccinated people to unvaccinated people… No study to date has examined the effect of the Pertussis vaccine and Influenza vaccine on pregnant women. This is a serious information gap for me. In addition, there is no prospective study following what happens to pregnant women who got vaccinated.” (Interviewee 10).

##### Ignoring vaccine-related injuries and reports on adverse effects

The issue of ignoring reports on vaccine-related injuries and side effects was raised by eight interviewees (1 hesitant and 7 anti-vaccination). Participants explained that the health authorities do not recognize the harm or injuries caused by vaccines. The interviewees also claimed that they know parents of vaccine-injured children whose lives changed after vaccinating their children.

“I know there's a lot of evidence from parents that can demonstrate their child's health status before and after vaccination, and I think it requires a thorough investigation and proper documentation…It shouldn't be dismissed or explained away by some excuse, like saying that autism often appears within the child's first year.” (Interviewee 2).

In addition, parents explained that there is currently no existing system in Israel that collects and processes reports or follow-ups regarding injuries and side effects caused by vaccines. As a result, knowledge about the extent of side effects in Israel and worldwide is inaccurate and unreliable. Therefore, the interviewees first demand recognition of vaccine-related injuries on the part of the authorities, and full transparency regarding the issue of side effects.

“The side effects of vaccines are underreported, and I think this is an important issue. The public doesn't get the real statistics on the adverse effects of vaccines…there is no reporting system. Therefore, the public doesn't know the incidence and prevalence of vaccine-related injuries and side effects.” (Interviewee 16).

#### Correcting misinformation and communicating uncertainty on social media

Facing misinformation and uncertainty is very common on social media. Ten interviewees (4 hesitant and 6 anti-vaccination) suggested that they face a great deal of misinformation and uncertainty on social media.

“I don't trust the social networks…they aren't a reliable source of information for decision making. For me, the media and social networks are a secondary source. First, I get information from a reliable source, and then from the social networks. But I don't make decisions based only on these networks.” (Interviewee 2).

Some of the parents perceive themselves as having low self-efficacy in identifying misinformation and finding accurate information. Others claim they can find accurate information, and know how and where to look for it. In addition, they described social media networks as unreliable sources of information. Moreover, they expressed difficulty in understanding and identifying uncertain and unclear information.

“Misinformation and uncertainty prevent me from deciding on complicated issues like vaccination. For example, if I read some information about a sports workout and I don't know whether it's correct or not, I won't do that workout.” (Interviewee 14).

### The second study objective: Parents' responses to the request to participate in academic research about their perceptions and attitudes regarding vaccination

During the preliminary process of the current study, 32 parents expressed initial consent to be interviewed. The study process and its goals were explained to these parents and interviews were scheduled. However, only 18 of the 32 interviews were conducted. The other 14 parents refused to participate in the study for several reasons. These reasons were recorded in detail in the field notes. Two main reasons for refusing to participate arose from analyzing the field notes.

#### Inappropriate time scheduled for the interview

Eight potential participants refused to be interviewed claiming that the scheduled time of the interview was inappropriate. At first, they postponed and rescheduled the interviews because of various constraints, such as an unexpected meeting, having to take care of their children, etc.

“Sorry, but… I have an unplanned meeting today. Can we postpose the interview till tomorrow?” (Potential participant 7).

The researchers contacted them again, more than once, until they eventually overtly refused to participate in the study.

#### Mistrust in academic institutions

The second reason for non–compliance of participating in the study was the hesitant and anti-vaccination parents' mistrust in academic research and its institutions. Academic institutions and researchers were perceived as untrustworthy by 7 out of 32 participants. They said they believe that academic institutions have hidden interests and agendas to comply with the pharma industry and health authorities.

“Corrupt people neglect our health. The vaccines are part of it, and the source of the funding is the pharmaceutical industry. Academia cooperates with the pharma industry. The fact of the matter is, that the pharma industry bought academia.” (Potential interviewee 2).

In addition, they think that most of the studies aim to promote vaccination and motivate more people to get vaccinated, instead of identifying the reasons that prevent the hesitant and anti-vaccination groups from vaccinating their children.

“But it's important for me to make it clear that we are skeptical toward studies aimed at improving communication with the public because, ultimately, we see that the goal of all these studies is to analyze the behavior of anti-vaccination people, and understand how to deal with these groups, instead of how to improve public health.” (Potential Interviewee 3).

Moreover, they suggest a lack of freedom to publish, and say that studies criticizing vaccines and vaccination cannot be published.

“I'm telling you, even if your study is excellent and really presents our attitudes, they won't allow you to publish it. Your study will not be published in any scientific journal.” (Potential Interviewee 2).

Some also claimed that hesitant and anti-vaccination parents' statements were manipulated in the media in order to damage their images. Therefore, they do not cooperate and refuse to be interviewed.

“Many parents' statements were manipulated in media interviews in which a trending edit was made to present them negatively. I wish you success in your research. I agree with you that it's important to bring a variety of opinions to academia and public debate.” (Potential interviewee 12).

Therefore, the potential participants asked the researchers questions regarding the aim of the study, its funding resources, and previous studies conducted by the researchers.

## Discussion

Vaccine hesitancy is one of the challenges that health organizations deal with in the public health sector. Most of the studies and meta-analyses in the literature have focused on developing strategies and campaigns to promote vaccination among hesitant and anti-vaccination subgroups (57, 58). On the other hand, there is a lack of studies that examined the perception and attitudes of parents from their perspective. Therefore, this study aims to identify the perceptions and attitudes of hesitant and anti-vaccination parents, the way they see themselves, how they perceive the health authorities, and the way they think the health authorities and the community perceive them during the measles outbreak in Israel.

The present study found that most of the interviewees, including anti-vaccination parents (who do not vaccinate their children), do not oppose vaccination, in general. In fact, they believe in the efficacy and importance of vaccines historically, and in the vital role of vaccines in eradicating many diseases. However, they oppose the vaccination process, including the way in which vaccines are promoted. From their perspective, both should be changed.

Therefore, the interviewees suggest a comprehensive, preventive, personalized medicine approach, based on tailoring a personalized vaccination schedule according to the child's health and background information. This approach is like a personalized medicine approach, which focuses on tailoring the best individually-suited treatment based on the person's unique clinical, genomic, and environmental information (59). In the field of prevention, personalized medicine, the literature indicates an emerging field of personalized vaccines. Theoretically, the idea of personalized vaccines—vaccines created to suit the individual—is based on a complex integration of the person's genetics, environmental and other factors, and the influence of his/her immune system's responses to vaccines (60). Personalized vaccinology is based on the concept of vaccinomics and adversomics. This approach explores the influence of genetic and non-genetic regulation on the variation of vaccine-induced immune responses at both the personal and population levels (61). Thus, the movement toward a personalized vaccines approach is likely to decrease adverse events rates and increase the public's confidence in vaccines (62). In addition, it is important to understand that hesitant and anti-vaccination parents are not anti-science per se. However, they are against the health authorities' lack of transparency regarding the efficacy and safety of vaccines. In addition, they critique the lack of public involvement in the decision-making process, such as personalized vaccinology and the separation of combination vaccines, which would make it possible to identify the unique side effects of each vaccine. In contrast to this finding, many studies consider anti-vaccination individuals as disseminators of misinformation and conspiracies, as well as science deniers (63).

One claim that arose in this study against the way vaccines are promoted is the lack of transparency regarding the issue of vaccine-related injuries and reports of adverse events. In this study, the interviewees claimed that health systems and organizations throughout the world ignore vaccine-related injuries. For example, there is no proactive system for reporting adverse events in Israel. In the United States, there is a Vaccine Adverse Event Reporting System (VAERS). This system is co-managed by the CDC and the FDA, which aim to monitor the safety of vaccines after they have been authorized or licensed for use by the FDA. However, this system has several limitations. VAERS is a passive reporting system, meaning that reports about adverse events are not automatically collected. In addition, a causal relationship cannot be established using information from VAERS reports alone. Moreover, the number of reports submitted to VAERS may increase in response to media attention and increased public awareness. Therefore, it is impossible to use VAERS data to calculate an adverse event incidence rate among the population (64, 65).

Another claim against the way in which vaccines are promoted which emerged from the interviews is the fact that vaccine-related injuries are not recognized by the health systems and authorities. Some governments (particularly those countries that mandate vaccination) have implemented no-fault vaccine injury compensation schemes, as a legal mechanism of resource for individuals experiencing adverse events following vaccination. Such schemes compensate a person or family who has experienced a serious injury or death caused by a vaccine, when no fault was found in the manufacturing or administration of the vaccine. The process of deciding whether compensation can be awarded requires systems to assess the causal link between the vaccine and the injury or death (66), yet currently no such systems are available.

In addition, in this study vaccine acceptance was found to be affected by distrust and lack of confidence in the safety and efficacy of vaccines and immunization, as well as in the healthcare system. Trust in the vaccine delivery system with all its components was found to be an important influencing factor in several explanatory models of vaccine hesitancy-related decision making. These models include the “3Cs” Model (17), “Vaccine Hesitancy Determinants Matrix” (2), and schema summary of a workshop on the cultural and religious roots of vaccine hesitancy in Canada (16). According to these models, trust in the system that delivers vaccines and the different types of information about vaccines mediate vaccination decisions (16, 18, 19). Therefore, health organizations should gain the trust of the parents in order to affect their decision regarding vaccination. In the empirical studies, trust was shown to have a positive effect on vaccination intention and uptake in most of the studies reviewed in a systematic review (17). Another study found that trust by laypeople in health systems and organizations depends on their performance. Low levels of confidence in the overall vaccination system and government management may lead to vaccine hesitancy and lower vaccination coverage (67, 68).

In other studies, transparent communication was found to reduce negative emotions and increase individuals' sense of respect toward the organization and the institution (69). In addition, the assumptions of this approach are based on the assumption that if health organizations provide complete and transparent information and address the emotional element, it will be more effective than when they deliver one-dimensional, partial responses that do not address the public's fears and concerns (70–73). Moreover, the SAGE found that poor communication can undermine acceptance and increase vaccine hesitancy and vaccine refusal (2). The literature on health communication emphasizes the importance of transparent information from health organizations in regard to addressing the public's worries and concerns (70, 74), in order to empower and increase the perceived self-efficacy of individuals and communities (75, 76). However, the findings of this study indicate that the health system and authorities do not provide the public with complete and transparent information. Instead, they provide partial information, ignore people's concerns, and do not answer their questions.

As a result, the parents, as emerges from this study, must search for information *via* other resources such as the news media and social networks. Therefore, it is important for health organizations to correct misinformation and practice full transparency, while addressing the emotional aspects of all the subgroups regarding vaccination (pro-vaccination, hesitant, and anti-vaccination).

In addition, this study largely clarifies why hesitant parents are perceived as being opposed to vaccines in principle, rather than those who simply oppose how the authorities promote vaccines in the public sphere. One explanation for this, as the study findings indicate, is their reluctance to participate in academic research. They claim that academic institutions and researchers have hidden agendas that serve the pharma industry and health authorities. As a result, a closed communication-loop situation is created. Paradoxically, hesitant and anti-vaccination parents choose to remain silent instead of expressing their attitudes and making their voices heard. This is because of the way they perceive the “bias” of all research conducted by academia. This perception should be further explored in future research.

### Study limitations

Although this is not a quantitative study using a representative sample of hesitant and anti-vaccination groups, the qualitative method enabled us to gain a deeper understanding of vaccine hesitancy and anti-vaccination phenomena, rather than a surface description of the attitudes of a large sample of the population.

It is important to note that this study was partly conducted during the COVID-19 pandemic. The development of this new vaccine has changed what falls under the umbrella of “vaccine-hesitancy”. An increasing number of parents and individuals chose to delay or refuse vaccination against COVID-19 (77). For example, vaccination acceptance in Israel dropped as additional doses of the vaccine were added (78). The vaccine hesitancy phenomenon has taken on a new meaning in which individuals who received all the vaccines—but have hesitant attitudes regarding the COVID-19 vaccine or refused to get vaccinated—were considered by their governments to be hesitant and anti-vaccination. Therefore, further studies should be conducted to study the specific implications of COVID-19 vaccination.

## Conclusion

### Recommendations for academic institutions

We recommend that academic institutions try to find new ways to make the voices of these groups heard (such as hesitant and anti-vaccination groups). Hesitant and anti-vaccination parents must also find a way to express their attitudes and perceptions. This is of great importance because, contrary to the prevailing stereotype of these groups as “conspiracy and misinformation disseminators” and “science deniers”, the current study found that they actually believe in science and in the vital role of vaccines in preventing diseases.

### Recommendations for health organizations

Health organizations need to engage in dialogue with hesitant and anti-vaccination groups. This dialogue should be based on a true and sincere interest in listening and understanding them, rather than trying to persuade them to change their minds.

In addition, health organizations should exercise caution in using a fear appeal strategy, which may adversely affect the public and lead to a lack of trust and cooperation. Instead, health organizations are advised to present all of the information, and openly address the public's fears and concerns. This may serve to increase the public's trust in health organizations and strengthen their reliability in the eyes of the public.

Therefore, the mutual collaboration of hesitant and anti-vaccination groups, academia, and health organizations may contribute to a better understanding of the barriers that prevent hesitant and anti-vaccination groups from getting vaccinated or vaccinating their children, thereby promoting public health.

## Data availability statement

The raw data supporting the conclusions of this article will be made available by the authors, without undue reservation.

## Ethics statement

The studies involving human participants were reviewed and approved by Faculty of Social Welfare and Health Sciences Ethics Committee for research with Human Subjects at the University of Haifa (Approval no. 421/17). The patients/participants provided their written informed consent to participate in this study.

## Author contributions

RH carried out this research as part of her PhD dissertation under the supervision of AG-E and GSM. RH conceptualized the study, reviewed the literature, conducted the data collection and analysis, written the manuscript, and took full responsibility for the study. AG-E provided input on the study conceptualization, data analysis, the first drafts, and the final version of the manuscript. GSM and PF-B critically reviewed the manuscript and helped shape the final version of the manuscript. All authors approved the final manuscript.

## Conflict of interest

The authors declare that the research was conducted in the absence of any commercial or financial relationships that could be construed as a potential conflict of interest.

## Publisher's note

All claims expressed in this article are solely those of the authors and do not necessarily represent those of their affiliated organizations, or those of the publisher, the editors and the reviewers. Any product that may be evaluated in this article, or claim that may be made by its manufacturer, is not guaranteed or endorsed by the publisher.
